# Social robots in care for older adults: a non-pharmacological option for the improvement of mental functioning?

**DOI:** 10.1192/j.eurpsy.2023.1997

**Published:** 2023-07-19

**Authors:** S. Tobis, J. Piasek, K. Wieczorowska-Tobis, A. Suwalska

**Affiliations:** 1 Department of Occupational Therapy, Poznan University of Medical Sciences; 2 Institute of Robotics and Machine Intelligence, Poznan University of Technology; 3 Geriatrics Unit, Chair of Palliative Medicine; 4 Department of Mental Health, Chair of Psychiatry, Poznan University of Medical Sciences, Poznan, Poland

## Abstract

**Introduction:**

With the rapid ageing of societies in Europe and worldwide, the issues of social functioning and mental well-being of older adults gain importance and call for effective care solutions. Among the non-pharmacological options, modern technologies are a promising direction. The use of humanoid social robots, at least in selected areas of care for community-dwelling older people, is one of the possibilities to cope with both their mental problems and the increasing shortage of qualified caregivers.

**Objectives:**

We thus investigated which prospective areas of care are scored best by older subjects and their professional caregivers.

**Methods:**

Opinions of older people (60+; no severe cognitive impairment), living in the community, and their professional caregivers about a robot in care for older adults were collected using the mixed-methodology Users’ Needs, Requirements and Abilities Questionnaire (UNRAQ), after a 90-150 minute interaction with the TIAGo robot (PAL Robotics, Spain).

**Results:**

The robot as a companion of an older person was scored better by older adults than caregivers (p<0.01). Similar results were obtained for the statements *The robot could decrease the sense of loneliness and improve the mood of the elderly person* (p<0.01), *The robot should detect the owner’s mood* (p<0.05), and *The robot should accompany the owner in everyday activities* (p<0.01).

**Conclusions:**

Our results, reflecting the opinions and preferences of various stakeholders, indicate a high general acceptance of a robot in care for older people. The indication of best-scored areas provides clues for the robot’s designers as well as those involved in the implementation of robotic solutions in care and their introduction into the lives of older adults.

**Disclosure of Interest:**

None Declared

